# Supplementary material for: Beneficial Effects of Mindfulness-Based Stress Reduction Training on the Well-Being of a Female Sample during the First Total Lockdown Due to COVID-19 Pandemic in Italy
Source: Int J Environ Res Public Health. 2021 May 21;18(11):5512. doi: 10.3390/ijerph18115512 (PMC8196575; doi:10.3390/ijerph18115512)
Supplement: Supplementary file 1 [file ijerph-18-05512-s001.zip › ijerph-1206355-supplementary.pdf]

Table S1. Baseline data (expressed as average score and standard deviation) and between-group statistical differences.

Test	Scale	MBSR Group	Control Group	t	*p*
**FFMQ**	Tot	24.4 (4.35)	26.8 (4.65)	−1.33	0.19
	OBS	23.9 (6.23)	26.4 (5.26)	−1.08	0.29
	DES	27 (7.02)	30.5 (7.70)	−1.19	0.25
	AWA	27.6 (6.20)	29.5 (6.50)	−0.77	0.45
	NJU	28.1 (5.90)	27.5 (6.36)	0.28	0.78
	NRE	15.8 (3.97)	20.4 (3.72)	−2.97	0.007*
**PWB**	Tot	49.2 (12.5)	66.4 (14.7)	−3.21	0.004*
	AU	8.33 (3.31)	9.18 (3.82)	−0.6	0.55
	EM	8.13 (2.23)	9.64 (3.59)	−1.32	0.2
	PG	8.07 (2.25)	12.1 (1.58)	−5.07	< 0.001*
	PR	8.47 (1.77)	12.6 (1.80)	−5.89	< 0.001*
	PL	8.13 (2.97)	11.5 (3.64)	−2.56	0.017*
	SA	8.07 (3.28)	11.4 (3.98)	−2.31	0.03*

Asterisks * ***p* < 0.05, ** *p* < 0.01, *** *p* < 0.001.** Abbreviations: FFMQ = Five Facet Mindfulness Questionnaire; PWB = Psychological Well-Being; OBS = Observing; DES = Describing; AWA = Acting With Awareness; NJU = Non-judging of inner experience; NRE = Non-reactivity to inner experience; AU = Autonomy; EM = Environmental Mastery; PG = Personal Growth; PR = Positive Relations; PL = Purpose in Life; SA = Self-Acceptance.

Table S2. Pre/post scores (expressed in the table as average and standard deviation) and results of the ANOVAs (detailed for the main effect of Group, the main effect of Time and the interaction effect between Group and Time).

Test	Scale	MBSR Group Pre	MBSR Group Post	Control Group Pre	Control Group Post	Group		Time		Group × Time	
		M (*SD*)	*M* (*SD*)	*M* (*SD*)	*M* (*SD*)	F	*p*	F	*p*	F	*p*
FFMQ	Tot	24.4 (4.35)	28.1 (3.98)	26.8 (4.65)	26.5 (6.46)	0.05	0.82	3.69	0.06	5.08	0.034*
	OBS	23.9 (6.23)	30.5 (4.27)	26.4 (5.26)	29.4 (6.19)	0.15	0.69	13.99	0.001*	1.97	0.17
	DES	27 (7.02)	29.5 (6.28)	30.5 (7.70)	26.6 (10.7)	0.009	0.92	0.29	0.59	7.1	0.014*
	AWA	27.6 (6.20)	27.7 (4.98)	29.5 (6.50)	28.5 (8.54)	0.37	0.55	0.12	0.72	0.21	0.65
	NJU	28.1 (5.90)	30.2 (7.49)	27.5 (6.36)	26.6 (8.54)	0.64	0.43	0.44	0.51	2	0.14
	NRE	15.8 (3.97)	22.7 (4.32)	20.4 (3.72)	21.5 (5.72)	1.28	0.26	20.2	< 0.001*	10.1	0.004*
PWB	Tot	49.2 (12.5)	61 (14.3)	66.4 (14.7)	62.5 (12.4)	3.18	0.08	10.9	0.003*	43.2	< 0.001*
	AU	8.33 (3.31)	8.87 (4.22)	9.18 (3.82)	9.45 (3.64)	0.27	0.61	0.48	0.49	0.05	0.82
	EM	8.13 (2.23)	9.2 (3.97)	9.64 (3.59)	9.64 (3.59)	0.62	0.44	1.03	0.32	1.03	0.32
	PG	8.07 (2.25)	11.2 (2.01)	12.1 (1.58)	11.7 (1.10)	11.9	< 0.001*	6.86	0.015*	10.94	0.003*
	PR	8.47 (1.77)	10.1 (2.33)	12.6 (1.80)	12.5 (2.25)	19.1	< 0.001*	4.87	0.037*	7.55	0.011*
	PL	8.13 (2.97)	11.2 (3.75)	11.5 (3.64)	9.45 (2.84)	0.4	0.53	1.34	0.25	30.33	< 0.001*
	SA	8.07 (3.28)	10.4 (3.04)	11.4 (3.98)	9.73 (3.93)	0.95	0.34	0.93	0.34	30	< 0.001*

Asterisks * ***p* < 0.05, ** *p* < 0.01, *** *p* < 0.001**. Abbreviations: FFMQ = Five Facet Mindfulness Questionnaire; PWB = Psychological Well-Being; OBS = Observing; DES = Describing; AWA = Acting With Awareness; NJU = Non-judging of inner experience; NRE = Non-reactivity to inner experience; AU = Autonomy; EM = Environmental Mastery; PG = Personal Growth; PR = Positive Relations; PL = Purpose in Life; SA = Self-Acceptance.

Table S3. PTGI scores (expressed as average score and standard deviation) and between-group statistical differences. AAQ-II scores expressed as average score and standard deviation) and within-group statistical differences.

Test	Scale	MBSR Group	Control Group	t	*p*
PTGI	Tot	36.6 (20)	29 (16.4)	1.03	0.31
	RO	10.7 (6.93)	5.91 (3.91)	20.71	0.049 *

	NP	8.27 (4.80)	7.27 (4.94)	0.51	0.61
	PS	7.33 (3.96)	5.55 (3.78)	11.59	0.26
	SC	1.93 (2.25)	1.09 (2.07)	0.97	0.34
	AL	6.27 (3.73)	6.18 (2.89)	0.06	0.95
AAQ-II	Tot	**PRE**	**POST**	**t**	***p***
		35.5 (6.23)	28.1 (9.74)	2.2	0.045*

Asterisks * ***p* < 0.05, ** *p* < 0.01, *** *p* < 0.001.** Abbreviations: PTGI = Post-Traumatic Growth Inventory; RO = relation to others; NP = new possibilities; PS = personal strength; SC = spiritual changes; AAQ-II = The Acceptance and Action Questionnaire-II**.**

Table S4. Model fit indices and unstandardized path coefficients for hypothesized mediation model.

**Predictor**	**Mediator**	**Outcome**	**Total Effect**	**Direct Effect**	**Indirect Effect**
			**SE–[95% CI]**	**SE–[95% CI]**	**SE–[95% CI]**
FFMQ pre-MBSR training	AAQ-II pre-MSBR training	PWB pre-MSBR training	1.6206–0.6570	1.9582–0.6515	−0.3376–0.3929
			[0.2011 ǀ 3.0401]*	[0.5384 ǀ 3.3780]*	[−1.1850–0.4261]
FFMQ post-MBSR training	AAQ-II post-MSBR training	PWB post-MSBR training	2.1831–0.6571	1.1924–0.5823	0,9907–0,4845
			[0.7632–3.6029]*	[-0.0766–2.4614]	[0.1377–2.0859]*

SE = Standard Error; CI = Confidence Interval; * $p < 0.05$.
